# Cortical basis for skilled vocalization

**DOI:** 10.1073/pnas.2122345119

**Published:** 2022-05-04

**Authors:** Christina M. Cerkevich, Jean-Alban Rathelot, Peter L. Strick

**Affiliations:** ^a^Neurobiology Department, University of Pittsburgh School of Medicine, Pittsburgh, PA 15261;; ^b^Systems Neuroscience Center, University of Pittsburgh School of Medicine, Pittsburgh, PA 15261;; ^c^Brain Institute, University of Pittsburgh School of Medicine, Pittsburgh, PA 15261;; ^d^Institut de Neurosciences de la Timone CNRS, Aix-Marseille Université, 13005 Marseille, France

**Keywords:** cerebral cortex, speech, laryngeal muscle, premotor areas

## Abstract

Marmosets display remarkable vocal motor abilities. Macaques do not. What is it about the marmoset brain that enables its skill in the vocal domain? We examined the cortical control of a laryngeal muscle that is essential for vocalization in both species. We found that, in both monkeys, multiple premotor areas in the frontal lobe along with the primary motor cortex (M1) are major sources of disynaptic drive to laryngeal motoneurons. Two of the premotor areas, ventral area 6 (area 6V) and the supplementary motor area (SMA), are a substantially larger source of descending output in marmosets. We propose that the enhanced vocal motor skills of marmosets are due, in part, to the expansion of descending output from these premotor areas.

## Significance

We examined the cortical control of a laryngeal muscle that is essential for vocalization in two monkey species that differ in their vocal motor skill. Our results suggest that enhancements in vocal skill are coupled to enlargements in the descending output from two premotor areas, ventral area 6 (area 6V) and the supplementary motor area (SMA). This result challenges the view that improvements in motor skills are due largely to changes in the output from the primary motor cortex.

Speech is a uniquely human form of communication which uses vocalization to express thoughts and feelings. Vocalization is built on the exquisitely coordinated control over respiration, phonation, and articulation. Historically, the enhanced vocal motor skills of humans have been attributed to alterations in the peripheral mechanisms for sound production (1, 2). However, recent studies of laryngeal biomechanics have ruled out this explanation (3). Instead, modifications in central neural circuits are the likely basis of the enhanced vocal abilities of humans (1). Here, we used a comparative approach to identify the adaptations in the cerebral cortex that provide a substrate for the enhanced vocal motor abilities of some monkeys.

Our experiments compared the areas of the cerebral cortex that are involved in the control of a laryngeal muscle in macaques and marmosets. We selected these two monkey species because of the striking differences in their vocal behavior. Macaque vocalization is generally limited to spontaneous utterances of acoustically simple calls which relate the animal’s emotional and motivational state (4). In the laboratory setting, it is difficult for researchers to elicit macaque vocalizations and for the monkeys to suppress spontaneous calls (5, 6). In contrast, marmosets readily vocalize in the laboratory setting. These monkeys naturally exhibit vocal turn taking with multiple back-and-forth exchanges that entrain to each other just as in human conversation (7–10). Marmosets can modulate the amplitude (11), timing (9, 11), and pitch (12) of their calls to compensate not only for physical noise but also for physical distance between conspecifics. Overall, marmosets demonstrate vocal skills and experience-dependent vocal production not observed in macaques (13–15).

To identify areas of the cerebral cortex that are involved in vocalization, we used retrograde transneuronal transport of rabies virus from the cricothyroid muscle. We selected the cricothyroid because it is the laryngeal muscle that is most specifically related to vocal motor control. The cricothyroid is an intrinsic laryngeal muscle that when active increases tension on the vocal folds (4). This muscle is unique in controlling vocal pitch while contributing little to other laryngeal functions, such as swallowing and airway regulation (4).

## Results

**Fig. 1.**
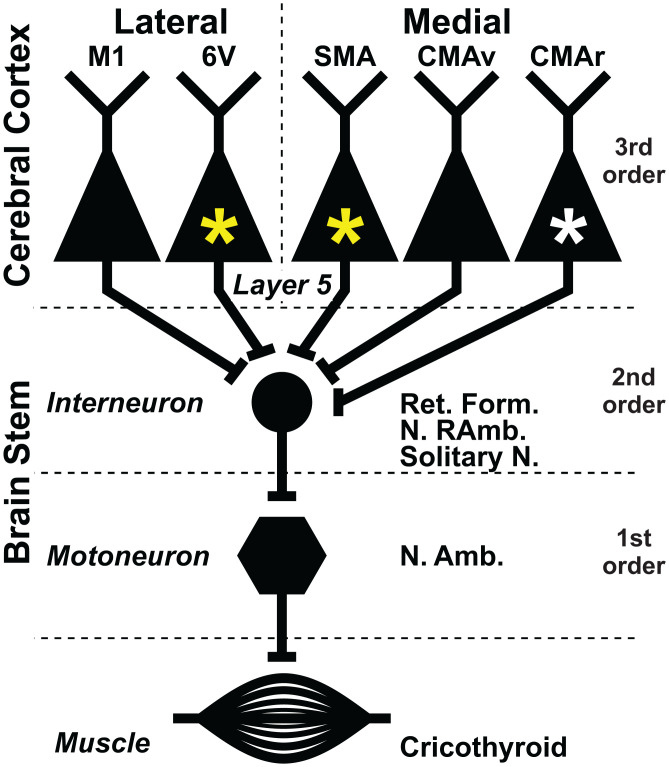
Multiple cortical motor areas participate in the generation and control of vocalization in macaques and marmosets. The third-order neurons in multiple areas of the cerebral cortex have disynaptic connections with laryngeal motoneurons. Two cortical areas, the SMA and area 6V (yellow asterisks), are enlarged in marmosets and may account for the superior vocal motor skills in these primates. A third cortical motor area, the CMAr (white asterisk), is enlarged in macaques and may mediate their reliance on vocalization with high emotional content. Ret. Form., Reticular Formation, N. RAmb, Retroambiguus nucleus, Solitary N., Solitary nucleus, and N. Amb., Ambiguus nucleus.

In both species of monkey, retrograde transport of rabies virus from the cricothyroid muscle infected the motoneurons that innervate the muscle. These “first-order” neurons are located in the nucleus ambiguus of the brainstem (Fig. 1, first order). As the survival time is extended (*SI Appendix*, Table S1), retrograde transneuronal transport of virus from these first-order neurons then infected “second-order” neurons at multiple sites including the retroambiguus nucleus, the nucleus of the solitary tract, and regions of the medullary reticular formation. All of these second-order sites are known to have monosynaptic connections with cricothyroid motoneurons (4) (Fig. 1, second order). Further extension of the survival time allows another stage of retrograde transneuronal transport to infect “third-order” neurons in the red nucleus, periaqueductal gray matter, and layer V within multiple areas of the cerebral cortex. These third-order neurons have disynaptic connections with cricothyroid motoneurons that are mediated by interneurons in the brainstem (Fig. 1, third order). We would like to emphasize that third-order neurons within a single animal, at both subcortical and cortical sites, were infected contemporaneously (*SI Appendix*, Fig. S1). This result is typical of experiments with rabies virus and reflects the fact that the slowest phase of the transneuronal process is the transsynaptic transfer of virus (16–19).

In two animals, transport infected a small number of “fourth-order” neurons outside of layer V in layers III and VI of the cerebral cortex (*SI Appendix*, Table S1). The results in these animals, including the overall distribution of infected neurons in layer V, did not differ from those with infection limited to third-order neurons. We also found infected neurons at central sites known to be involved in autonomic control, like subgenual cingulate cortex (20). It is likely that this labeling is mediated by autonomic efferents that innervate glands and blood vessels embedded in the cricothyroid muscle (21–25). Although the activity of these autonomic efferents can affect voice quality, these efferents are not directly involved in causing contraction of the cricothyroid muscle (21–25).

It is noteworthy that we found no evidence of second-order neurons infected in layer V of the motor cortex in either monkey. This result supports prior reports that the motoneurons in the nucleus ambiguus lack monosynaptic connections from output neurons in the primary motor cortex of the monkey (26). Specifically, our results support the conclusion that cricothyroid motoneurons in the macaque and the marmoset lack corticomotoneuronal connections. Altogether, these patterns of virus transport are fully consistent with the results from neuroanatomical experiments using conventional tracers (4).

**Fig. 2.**
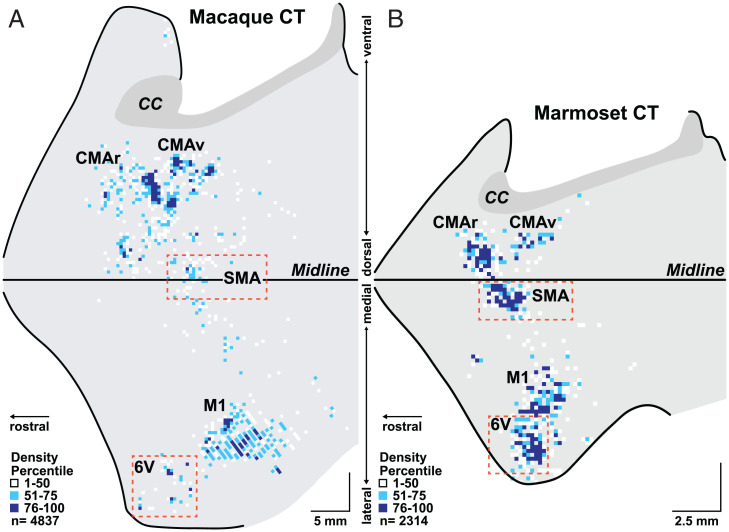
Origin of cortical output to a laryngeal muscle in a macaque and a marmoset. We injected rabies virus into the same laryngeal muscle (cricothyroid [CT]) in a macaque (*A*) and a marmoset (*B*). We set the survival time to allow retrograde transneuronal transport of the virus to output neurons in layer V of the cerebral cortex. In these maps, the medial wall of the hemisphere is reflected upward. In the macaque, the anterior bank of the central sulcus was reconstructed separately and joined to the lateral surface to aid in the comparison of results from the marmoset, which lacks a central sulcus. Other sulci on the lateral surface of the macaque have not been unfolded and are not displayed. (*SI Appendix*, Fig. S2 displays the same macaque results with sulcal markers present.) Colored squares indicate the density of the infected neurons in bins throughout the cerebral cortex (macaque bins, 400 × 400 µm; marmoset bins, 200 × 200 µm). Bins with one infected cell are not displayed. Dashed rectangles enclose area 6V and SMA regions that display expanded cortical output in marmosets compared with macaques. Note scale differences between macaque and marmoset maps. (Scale bars, 5 mm [macaque] and 2.5 mm [marmoset].) CC, corpus callosum. Dark gray shading indicates the corpus callosum; thick lines indicate the midline and edges of the frontal lobe block.

**Fig. 3.**
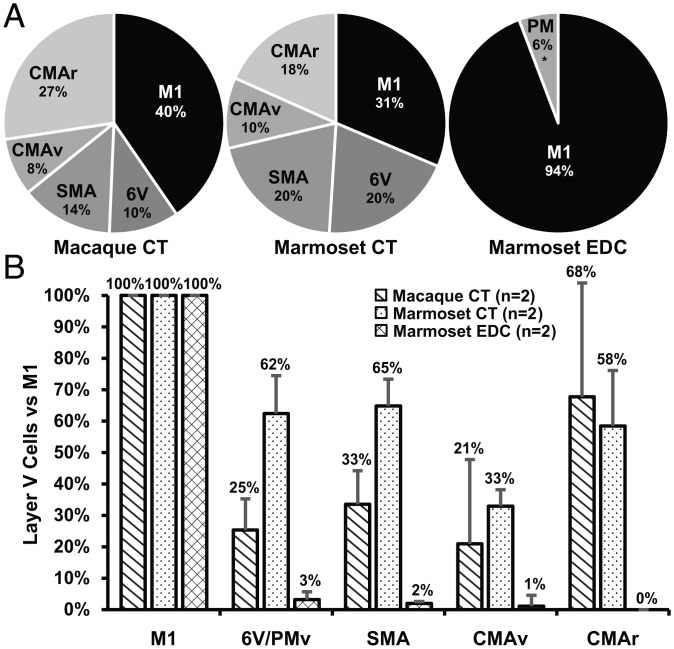
Quantitative analysis of cortical outputs to the motoneurons of a laryngeal (CT) and a hand (EDC) muscle in macaques and marmosets. (*A*) Percentage of infected neurons in layer V within each of the cortical motor areas following retrograde transneuronal transport of rabies virus from the CT and EDC muscles of macaques and marmosets. (*B*) The number of infected neurons in layer V within each of the cortical motor areas is normalized to that found in M1 of each animal. The error bars represent the SD of the results from *n* = 2 for each experiment.

As noted above, third-order neurons are infected in layer V of multiple cortical motor areas in the frontal lobe of both the macaque and marmoset (Fig. 2 and *SI Appendix*, Figs. S2–S5). Thus, all of these areas have disynaptic connections with motoneurons. These cortical motor areas include the primary motor cortex (M1) and four premotor areas in the frontal lobe. One of the premotor areas is on the lateral surface of the hemisphere: ventral area 6 (area 6V). The other three premotor areas are on or near the medial wall of the hemisphere: the supplementary motor area (SMA), ventral cingulate motor area (CMAv), and rostral cingulate motor area (CMAr). A quantitative analysis shows that the majority of the layer V neurons are located outside of M1 in the premotor areas (macaque, 60%; marmoset, 69%) (Fig. 3*A*). Thus, the cortical control of vocalization in macaques and marmosets is mediated by parallel pathways originating from multiple motor areas in the frontal lobe. Furthermore, the premotor areas have disynaptic access to motoneurons just like M1.

We examined the number of output neurons in layer V of each premotor area to assess their relative contribution to vocal motor control. This analysis showed that the disynaptic output to laryngeal motoneurons from area 6V and the SMA is significantly greater in the marmoset than in the macaque (Fig. 3*B*). For example, the number of output neurons in area 6V of the marmoset is 62% of that in M1, whereas the number of output neurons in area 6V of the macaque is only 25% of that in M1. Similarly, the number of output neurons in the SMA of the marmoset is 65% of that in M1, whereas the number of output neurons in the SMA of the macaque is only 33% of that in M1. Thus, expansions in descending output from two premotor areas, area 6V and the SMA, correlate with the enhanced vocal abilities of marmosets.

One could argue that our results reflect a more general species difference between macaques and marmosets in the organization of the premotor areas. If this were the case, then the relative expansion of area 6V and the SMA seen in marmosets for laryngeal muscles should also be present for the muscles that control other body parts. To test this explanation, we examined cortical labeling in the marmoset after retrograde transneuronal transport of rabies virus from a hand muscle, the extensor digitorum communis (EDC). We selected EDC because this muscle is essential in all primates for manual actions like gripping objects. EDC extends and spreads the fingers as part of the act of grasping (27). We also selected EDC because, in other monkeys, it is a major target of cortical output (28) and we have previously used transneuronal transport of rabies virus to determine the cortical neurons that control this muscle in macaques (29).

**Fig. 4.**
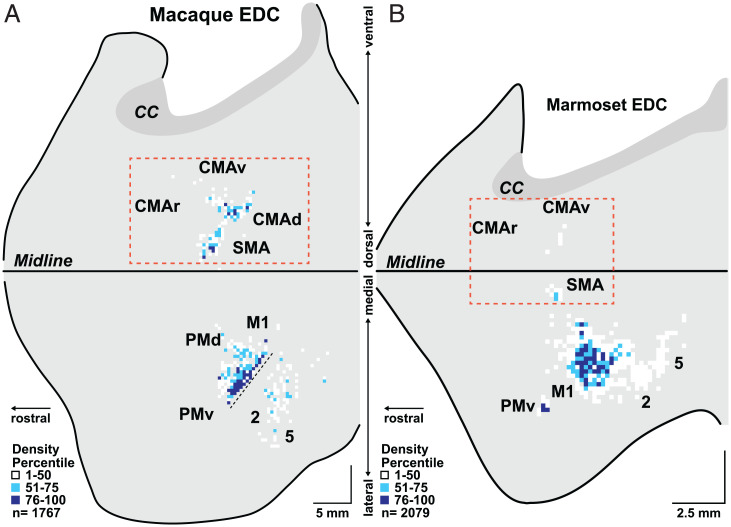
Origin of cortical output to a hand muscle in a macaque and a marmoset. We injected rabies virus into the same hand muscle (EDC) in a macaque (*A*) and a marmoset (*B*). We set the survival time to allow retrograde transneuronal transport of the virus to output neurons in layer V of the cerebral cortex. The distribution of the infected neurons is displayed on flattened maps of the frontal cortex (for figure and scale details, see Fig. 2). Dashed rectangles enclose the CMAr, CMAv, CMAd, and SMA regions that display expanded cortical output in macaques compared with marmosets. The fine dashed line in M1 indicates the region of the central sulcus that is not opened in this diagram. The anterior bank of the sulcus at this site contains a region of M1 that has layer V neurons that make monosynaptic connections with EDC motoneurons. The macaque EDC data in *A* have been remapped and adapted from Strick et al. (29). Cortical areas: CMAd, dorsal cingulate motor area; PMd, dorsal premotor motor area.

The overall distribution of infected neurons in the frontal lobe of marmosets following transport of rabies virus from EDC is strikingly different from that following transport from the cricothyroid (compare Fig. 4 and *SI Appendix*, Figs. S6 and S7 with Fig. 2 and *SI Appendix*, Figs. S4 and S5; see also Fig. 3). For example, in the marmoset, the overwhelming majority (94%) of the cortical neurons infected following virus transport from EDC is located in M1 (Figs. 3 and 4). Only small clusters of infected neurons are located in the ventral premotor area (PMv; 3%) or in the SMA (2%), and a few isolated infected neurons are scattered on the medial wall of the hemisphere in the region of the CMAv. In fact, area 5L in the posterior parietal cortex of the marmoset contains more infected neurons than all the premotor areas together. Clearly, the premotor areas of the marmoset are not a major source of disynaptic signals to control EDC motoneurons. This result is quite different from the pattern of infection observed following retrograde transneuronal transport of rabies from EDC of macaques. In this primate, the premotor areas are a substantial source of disynaptic output to hand motoneurons (Fig. 4) (29). Thus, the paucity of disynaptic output from the premotor areas to marmoset hand motoneurons correlates with the relatively limited hand skills of these primates compared with macaques (30, 31). In contrast, the expansion of disynaptic output from area 6V and the SMA to laryngeal motoneurons may provide a neural substrate for the superior vocal motor skill of marmosets.

## Discussion

Others have proposed that the vocal skill necessary for speech depends on the addition of a monosynaptic connection between layer V neurons in the “laryngeal motor cortex” and motoneurons in the nucleus ambiguus (4, 26, 32). The presence of a corticomotoneuronal connection from layer V neurons in the hand area of M1 to motoneurons has been linked to the enhanced manual dexterity of humans, great apes, and some monkeys (29, 33). However, neither macaques nor marmosets have corticomotoneuronal connections with laryngeal motoneurons. Instead, our results emphasize the importance of disynaptic control of motoneurons by output neurons located in multiple cortical motor areas which lie outside of M1 (Fig. 1).

Students of motor control are often left with the impression that M1 serves as a single “upper motoneuron” for the central generation and control of movement. Although it is correct that M1 is a major source of corticospinal and corticobulbar neurons, it is by no means the sole source (29). In fact, our findings about disynaptic control of a vocal muscle indicate that the total number of output neurons located in the premotor areas exceeds the number in M1 (Fig. 3*A*). In addition, we show that the number of output neurons in the laryngeal representation of several individual premotor areas approaches that in M1 (Fig. 3*B*). Thus, the cortical control of vocalization in both macaques and marmosets is accomplished by parallel pathways descending not only from M1 but also from multiple premotor areas in the frontal lobe (Fig. 1).

Our findings indicate that an expansion of the descending output from the laryngeal representation in two specific premotor areas in the frontal lobe (i.e., area 6V and the SMA) correlates with the enhanced vocal capabilities of marmosets compared with macaques. Thus, an important conclusion from our study is that enhanced skill, in this case in the vocal domain, may depend on expansions in disynaptic connections to laryngeal motoneurons from the premotor areas in the frontal lobe, rather than alterations in M1 output. This conclusion is further supported by the striking differences we observed in the cortical origin of disynaptic outputs to marmoset laryngeal and hand motoneurons. The rudimentary manual dexterity of marmosets (30, 31) parallels the relative absence of disynaptic projections to EDC motoneurons from cortical areas outside of M1 in these monkeys. Thus, our results support the proposal that an expansion in descending output from premotor areas in the frontal lobe provides a neural substrate for enhanced motor skill (29).

There has been considerable speculation that skilled hand movements and vocalization coevolve and share a common neural substrate (34). The cortical control of vocalization and hand movements in marmosets presents a clear counterexample. As noted above, the vocal skills of marmosets far exceed their hand skills. Similarly, the expansions we see in the cortical areas concerned with vocalization are not matched by comparable changes in cortical areas concerned with manual dexterity. Thus, vocal skill, hand skill, and their neural substrates are not linked in these nonhuman primates.

There is a large and confusing literature on the involvement of the different cortical motor areas in vocalization. Space limitations preclude us from reviewing the entirety of this literature here. However, several general conclusions emerge from our analysis of it. There is evidence that each premotor area makes a unique contribution to vocal control. In humans, area 6V appears to be important for encoding the movements of the larynx, tongue, jaw, and lips to meet a vocalization goal and produce the desired sound (35–37). Similarly, area 6V in marmosets may be essential for their ability to shape and control the elements of their individual calls as they rapidly adjust vocal amplitude and pitch (11, 12, 38). On the other hand, the SMA is thought to be involved in sequencing, timing, and the initiation of complex human vocalization and speech (39–43). This cortical area in marmosets may be essential to their ability to sequence the syllables and control the complex timing structure of their multisyllabic calls (44). The expanded vocal repertoire of marmosets fits with an enlargement in the descending output from both area 6V and the SMA.

Another premotor area in the frontal lobe, the CMAr, has historically been linked to the expression of emotion and autonomic control (4, 20, 45). Macaques largely use vocalization to signal their emotional status, and thus the expanded CMAr in this species reflects its greater reliance on this type of vocalization. Jürgens and others believed that the output from the CMAr was mediated by connections with the periaqueductal gray which then had disynaptic connections with laryngeal motoneurons (4). This would translate into a pathway from the CMAr to laryngeal motoneurons that requires a chain of four synaptically connected neurons. However, our data indicate that the pathway from the CMAr to laryngeal motoneurons, as well as those from area 6V, the SMA, and CMAv, requires a chain of only three synaptically connected neurons (i.e., third order, Fig. 1). In other words, all the cortical motor areas involved in the control of vocalization have disynaptic access to laryngeal motoneurons.

Our basic proposal is that alterations at the level of descending output from multiple cortical motor areas explain the enhanced vocal abilities of marmosets compared with macaques. This does not preclude improvements in vocal motor control due to alterations at other sites of the neuroaxis, such as the basal ganglia and cerebellum. In fact, we have argued that these subcortical structures are part of an integrated network with the cerebral cortex (46). Thus, it would not be surprising to see alterations at other nodes in the network leading to improvements in vocal performance. Even so, our results support the proposal that the existence and expansion of descending output from the premotor areas in the frontal lobe are a major macroarchitectural change that provides the neural substrate for enhanced motor skill (29).

## Materials and Methods

We used retrograde transneuronal transport of rabies virus (CVS-N2c; M. Schnell, Thomas Jefferson University, Philadelphia, PA) to reveal the cortical areas that influence different muscles in macaques and marmosets. We saw no differences in the way the N2c strain infected marmoset and macaque neurons (16). For example, over the survival periods used in these experiments, the infections with rabies virus were confined to neurons, and we saw no evidence of glial infection, cell lysis, or other visible tissue damage (*SI Appendix*, Fig. S1). Indeed, it is well-known that infection with rabies virus results in surprisingly limited pathology even in the brains of subjects at terminal stages of the disease (47). Furthermore, during the survival times used in these experiments, both marmosets and macaques infected with the N2c strain remained largely free of symptoms.

We injected rabies virus into the right cricothyroid muscle in four adult macaques (three *Macaca mulatta*; one *Macaca fascicularis*) and three adult marmosets (*Callithrix jacchus*). In addition, we injected rabies virus into the right EDC muscle in two marmosets. The specifics of each animal, virus batch, injection, survival time, and cortical distribution of infected neurons are presented in *SI Appendix*, Table S1. In addition, we reanalyzed previously published data on the cortical areas influencing EDC in macaques (48, 49).

All procedures were in accordance with the Association for Assessment and Accreditation of Laboratory Animal Care and the NIH *Guide for the Care and Use of Laboratory Animals* (50). The University of Pittsburgh’s Institutional Animal Care and Use and Biosafety committees approved all experimental protocols. Biosafety practices conformed to biosafety level 2+ regulations outlined in *Biosafety in Microbiological and Biomedical Laboratories* (51). Procedural details for handling virus and virus-infected animals have been published (16, 17).

### Surgical Procedures.

All surgical procedures were performed under general anesthesia and aseptic conditions. Monkeys were fasted, initially anesthetized with ketamine hydrochloride (macaques: 10 to 20 mg/kg, intramuscularly [IM]; marmosets: 20 to 40 mg/kg, IM), and intubated or masked and maintained on 0.25 to 5% isoflurane for the duration of the procedure. Each animal received appropriate presurgical cefa-class antibiotics (25 to 75 mg/kg, IM) and analgesics (macaques: buprenorphine, 0.01 to 0.03 mg/kg, IM; marmosets: meloxicam, 0.1 to 0.2 mg/kg, IM). Monkeys received fluids throughout the procedure (macaques: saline, 2 to 7 mg⋅kg^−1^⋅h^−1^ intravenously [IV] or 5% lactated Ringer’s solution, 2 to 10 mL⋅kg^−1^⋅h^−1^; marmosets: lactated Ringer’s solution, 6 to 20 mL⋅kg^−1^⋅h^−1^, IV or subcutaneously). We monitored heart rate, blood oxygen saturation, end tidal carbon dioxide, and respiration rate until the animal was sufficiently recovered from anesthesia. We used a circulating water heating pad or a Bair Hugger to maintain the monkey’s temperature at 36 to 38 °C. A small incision in the skin and careful dissection of any overlying muscles exposed target muscles. Each muscle was identified by its origin, insertion, and electrical stimulation (0.2-ms pulses at 25 Hz for 1 s, at a maximum intensity of 15 V). We used a Hamilton microsyringe with a 30-gauge needle to place multiple small injections of rabies virus into each muscle (for details, see *SI Appendix*, Table S1). Following each injection, we held the syringe in place for 1 min and blotted the injection site with a sterile cotton swab upon removal of the injection needle to prevent leakage. The wound was sutured in layers. Upon recovery from anesthesia, each monkey was treated with the appropriate analgesic (macaques: a single dose of buprenorphine, 0.01 to 0.03 mg/kg, IM; marmosets: a single dose of buprenorphine, 0.005 to 0.01 mg/kg, IM, followed by additional doses of buprenorphine or meloxicam, 0.1 to 0.2 mg/kg, orally, if needed), and then placed in isolated housing for virus-infected animals.

At the end of the survival period (*SI Appendix*, Table S1), each monkey was anesthetized (ketamine, 20 to 25 mg/kg, IM), followed by sodium pentobarbital (40 mg/kg, intraperitoneally), and perfused transcardially with a three-step procedure. The perfusates included 1) 0.1 M phosphate buffer, 2) 10% (volume [vol]/vol) phosphate-buffered formalin, and 3) 10% phosphate-buffered formalin with 10% (vol/vol) glycerol added. After perfusion, we removed the brains and cut them into four blocks: 1) the cerebral cortex and diencephalon, 2) brainstem, 3) cerebellum, and 4) cervical spinal cord. The blocks were postfixed for up to 2 wk in 10% (vol/vol) phosphate-buffered formalin with 20% (vol/vol) glycerin at 4 °C.

### Histological Procedures.

We cut each tissue block in serial frozen sections (50 µm) in the coronal plane. Every 10th section of the cerebral cortex and brainstem blocks and every 20th section of the spinal cord were stained with cresyl violet to reveal cytoarchitecture. To identify virus-infected neurons, we processed every free-floating tissue section according to the avidin-biotin peroxidase method (Vectastain ABC Kit PK-4002, Vector Laboratories). Rabies antigen was detected using a mouse monoclonal antibody directed against the rabies virus phosphoprotein (M957, diluted 1:300; supplied by A. Wandeler, Animal Disease Research Institute, Ottawa, ON, Canada) (16, 17, 52). Each section was mounted on gelatin-coated glass slides, air-dried, and coverslipped with Cytoseal.

### Data Collection and Analysis.

We charted the location of infected neurons, gray–white boundaries, and section outlines with an Olympus microscope using a computer-based charting system (MD Plot 4, Minnesota Datametrics). This system relies on linear optical encoders that are coupled to *X*–*Y* movements of the microscope stage. The same system was used to indicate layer V and confirm anatomical borders of cortical areas. We also examined the brainstem, cerebellar, and spinal cord sections to confirm the order of virus transport.

We then used custom software (ReconWin, Great Island Software) to combine and align digitized sections, mark landmarks like sulci, and digitally unfold the cortex to create reconstructed flat maps of infected neurons in the cerebral cortex. The same software enabled us to overlap maps and compare the consistency results between animals. To determine the relative contribution of each cortical motor area, we counted the number of infected neurons in the cortical areas of individual cases using ReconWin. The total number of neurons in each area was corrected to equal the number of cells if every section had been plotted (i.e., multiplied by 2 for cases where cells were plotted on every other section or by 4 for cases where cells were plotted on every fourth section). We then added the number of cells in each area across matched cases to create an aggregate distribution for each target muscle. We performed a χ^2^ goodness-of-fit test (*P* ≤ 0.001) to determine if the distribution of labeled cells in M1 and premotor areas differed for each muscle injected. We then used a critical ratio test for independent proportions (*P* ≤ 0.001, Bonferroni-corrected for multiple comparisons) to compare the proportion of infected cells in each area across cases.

We used a NanoZoomer S60 (Hamamatsu Photonics) to capture images of rabies virus–infected neurons (*SI Appendix*, Fig. S1) in select sections after charting. Slides were scanned at nine depths with a numerical aperture of 0.7. We then used custom software to compress the *z* stack into a single focused image. We used NDP.view2 (Hamamatsu Photonics) to view and export compressed images and Photoshop (Adobe Systems) to crop, desaturate, and adjust image curves.

## Supplementary Material

Supplementary File

## Data Availability

All study data are included in the article and/or *SI Appendix*. All other data are available upon request from the corresponding author.
