# Supplementary material for: Cortical basis for skilled vocalization
Source: Proc Natl Acad Sci U S A. 2022 May 4;119(19):e2122345119. doi: 10.1073/pnas.2122345119 (PMC9171651; doi:10.1073/pnas.2122345119)
Supplement: Supplementary File [file pnas.2122345119.sapp.pdf]

PNAS
www.pnas.org

Supplementary Information for
Cortical basis for skilled vocalization

Christina M. Cerkevich, Jean-Alban Rathelot, Peter L. Strick

Corresponding author: Peter L. Strick
Email: strickp@pitt.edu

This PDF file includes:

Figures S1 to S7
Table S1

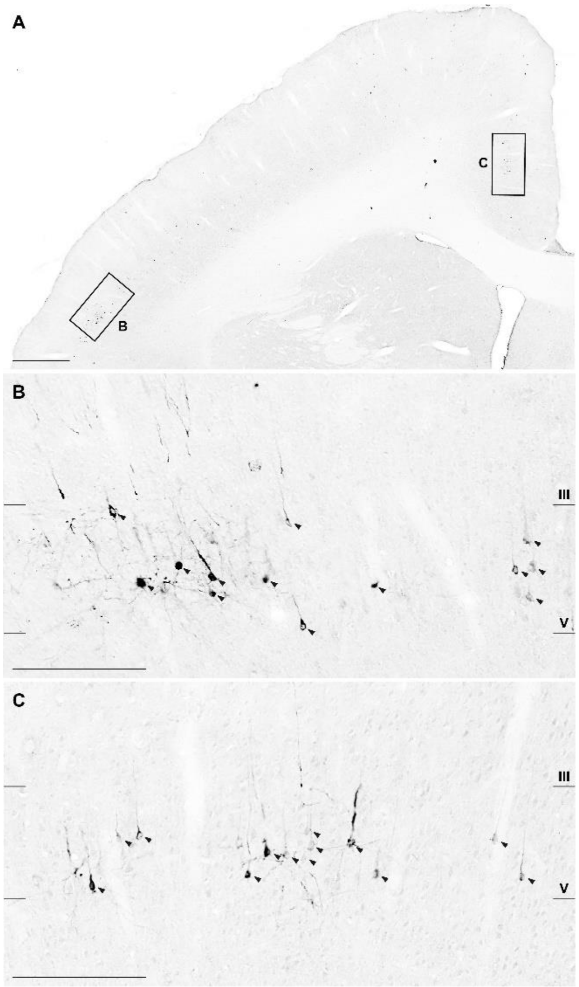

Fig S1. Layer V neurons infected with rabies virus following transneuronal transport from marmoset CT. Multiple cortical areas contain infected neurons limited to layer V at survival times long enough to label third-order neurons (see Fig. 1). Here we illustrate infected neurons in two of those cortical areas on a single section. The small boxes in A illustrate the location of the enlargements in B and C. B. Ventral area 6 (6V). C. Cingulate motor area, ventral (CMAv). The small arrowheads in B and C mark the soma of each infected neuron. Tick marks at the edges of B and C indicate the upper and lower border of layer V. Scale in A is 1 mm. Scales in B and C is 250 µm.

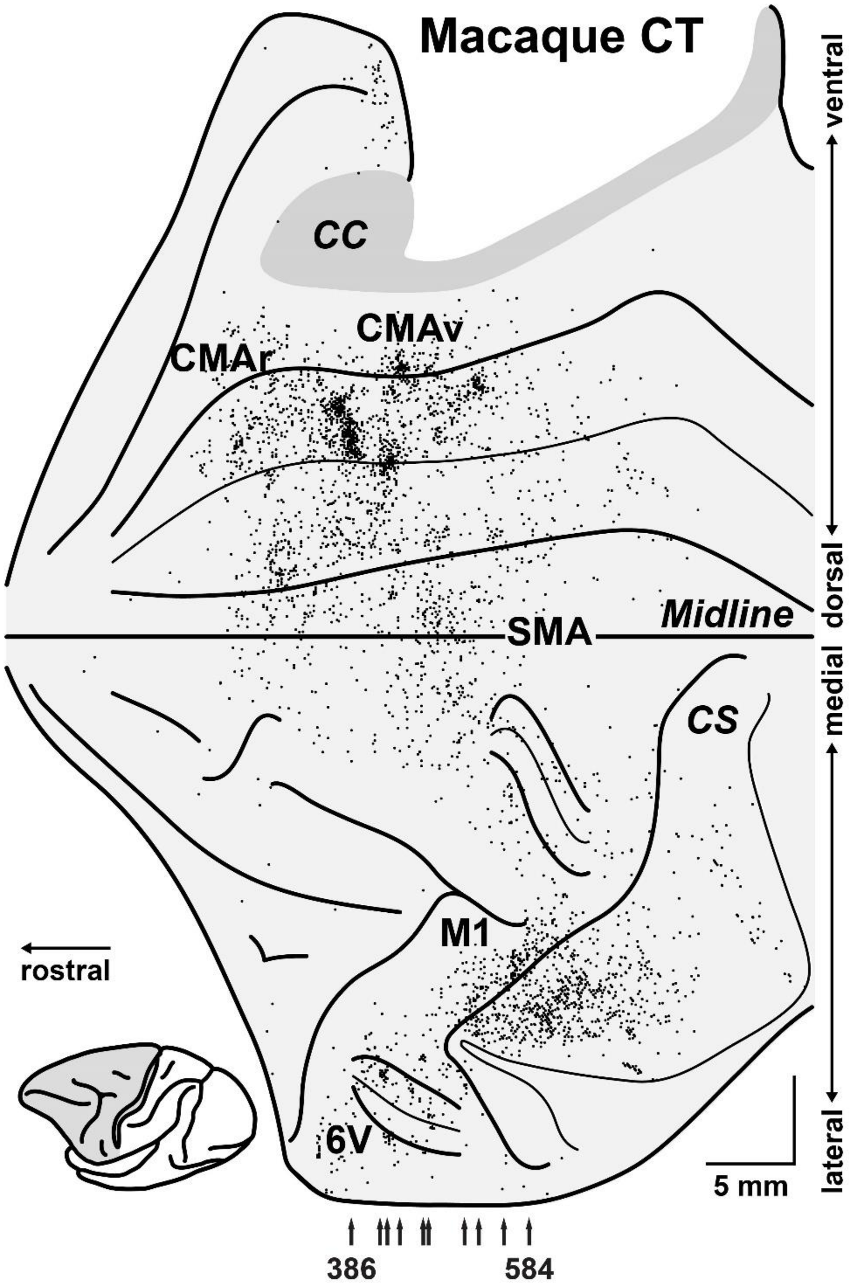

Fig. S2. Cortical map of output to macaque CT. The locations of infected neurons are indicated by small dots. The density map displayed in Fig. 2a is based on this raw data. The small arrows and numbers at the bottom of the diagram indicate the planes of the sections displayed in Fig. S3. (For details, see the legend of Fig. 2.)

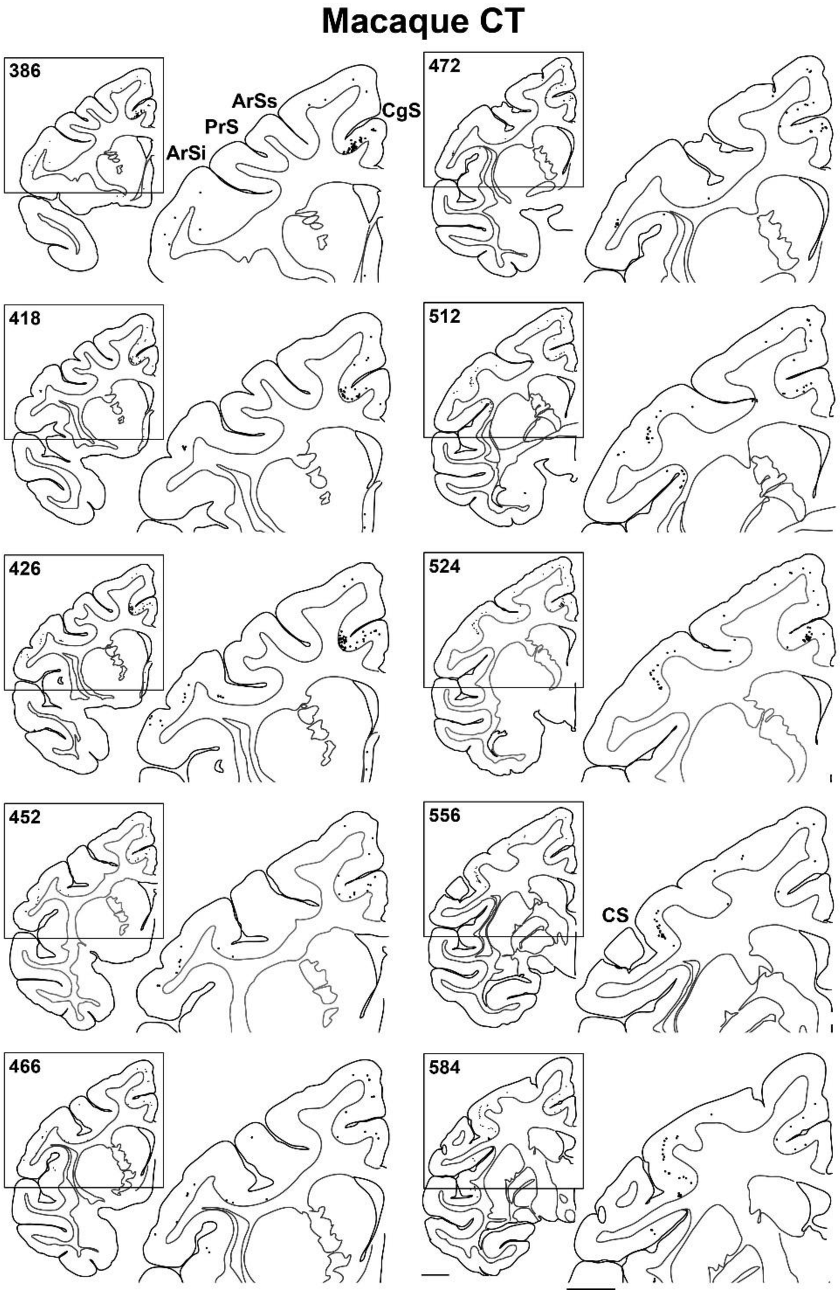

Fig. S3. Cells of origin of cortical output to macaque CT. Small dots indicate the locations of infected neurons found on single sections through the macaque frontal lobe. The planes of these sections are indicated by the small arrows and numbers in Fig. S2. Scale is 2.5 mm. ArSs- arcuate sulcus, superior limb; ArSi- arcuate sulcus, inferior limb; CgS- cingulate sulcus; CS- central sulcus; PrS-principal sulcus.

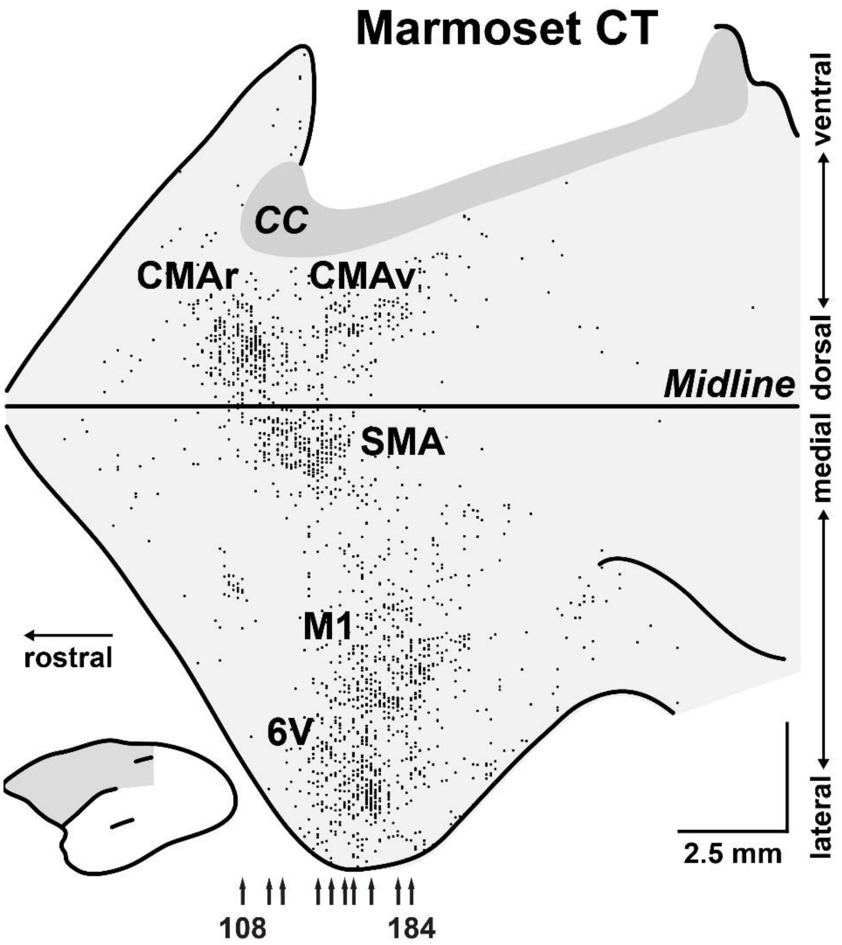

Fig. S4. Cortical map of output to marmoset CT. The locations of infected neurons are indicated by small dots. The density map displayed in Fig. 2b is based on this raw data. The small arrows and numbers at the bottom of the diagram indicate the planes of the sections displayed in Fig. S5. (For details, see the legend of Fig. 2.)

Marmoset CT

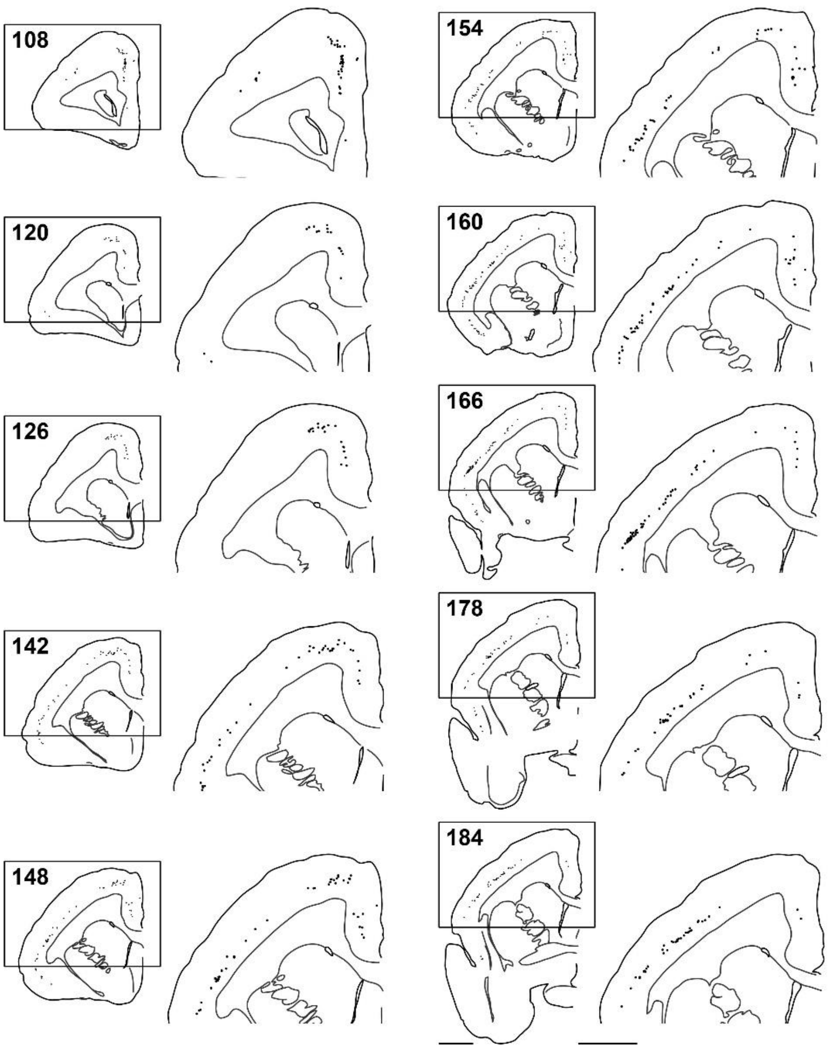

Fig. S5. Cells of origin of cortical output to marmoset CT. Small dots indicate the locations of infected neurons found on single sections through the marmoset frontal lobe. The planes of these sections are indicated by the small arrows and numbers in Fig. S4. Scale is 2.5 mm.

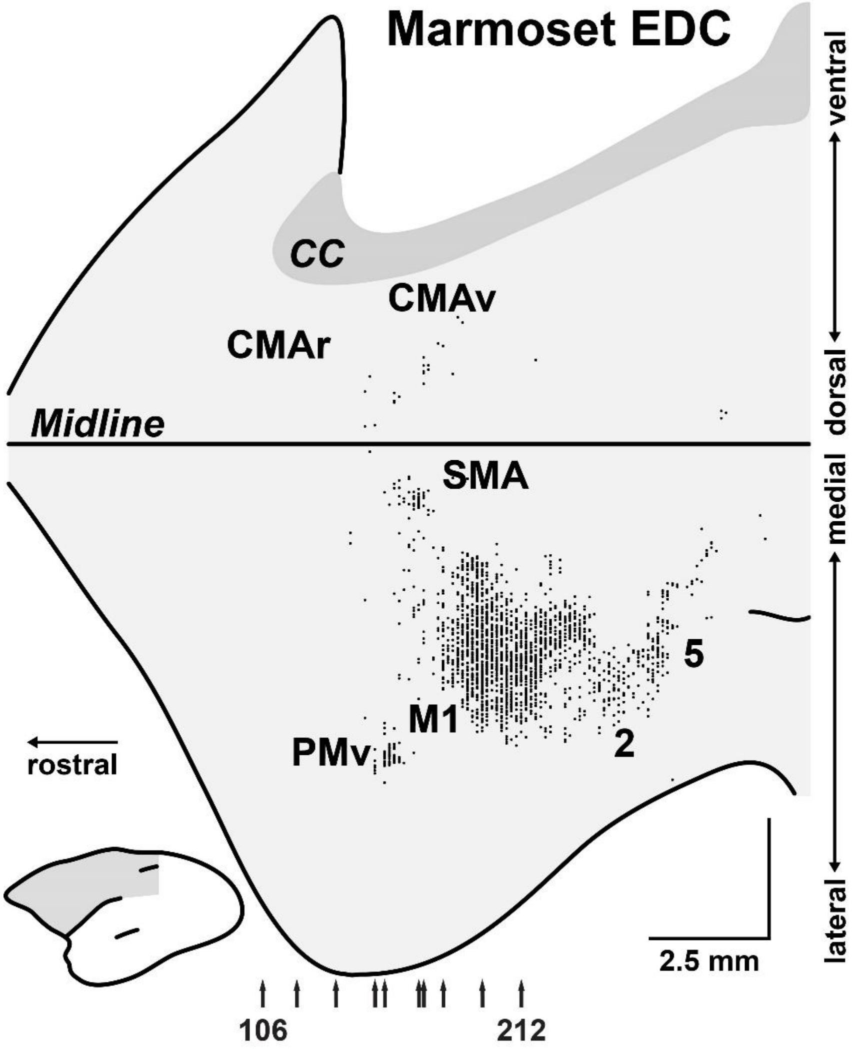

Fig. S6. Cortical map of output to marmoset EDC. The locations of infected neurons are indicated by small dots. The density map displayed in Fig. 4b is based on this raw data. The small arrows and numbers at the bottom of the diagram indicate the planes of the sections displayed in Fig. S7. (For details, see the legend of Fig. 2.)

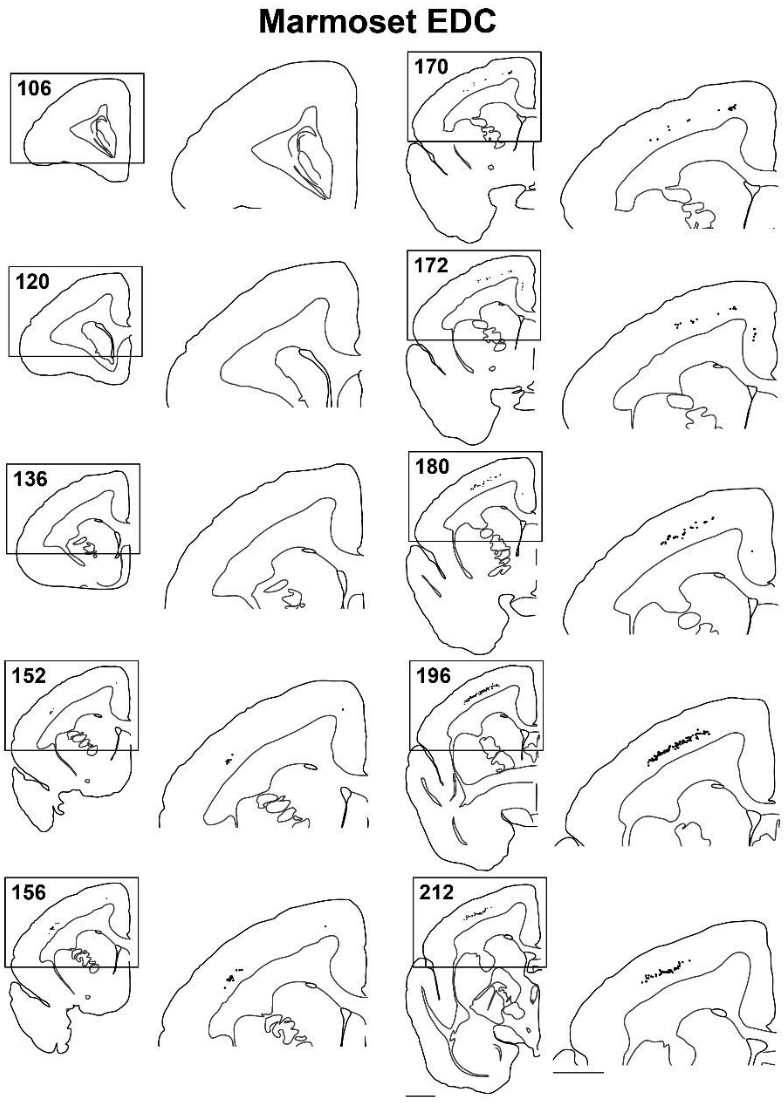

Fig. S7. Cells of origin of cortical output to marmoset EDC. Small dots indicate the locations of infected neurons found on single sections through the marmoset frontal lobe. The planes of these sections are indicated by the small arrows and numbers in Fig. S6. Scale is 2.5 mm.

Table S1. Experimental information. Monkey case number and sex with the *M. fascicularis* marked with an asterisk; animal weight (kg) and age (years); muscle injected (CT, cricothyroid; EDC, extensor digitorum communis); rabies virus batch; virus concentration (pfu/ml); volume injected (µl); survival time (hours); percentage of injected neurons in Layer V. Note1: 0% indicates that no neurons were infected in any area of the cerebral cortex. In all instances, this was due to the survival time being too short to infect third-order neurons. However, the survival time in these cases was long enough to infect many second-order neurons at other sites. Note2: transneuronal transport rates vary between batches of virus. Thus, survival time alone is not sufficient to determine the "order" of transport.

Macaque	*Weight/ Age (kg/yrs)*	*Muscle*	*Batch*	*Conc. (pfu/ml)*	*Volume (µl)*	*Survival (Hours)*	*Layer V (%)*
JA71 (m)	4.6/4.5	CT	5/24/13	9×10^8	30	115	84.1%
JA68 (m)	4.32/4	CT	5/24/13	9×10^8	30	107	0%
CC09 (f)*	3.2/3	CT	16-70	1×10^9	39	84	89.4%
CC06 (m)	8.3/4.5	CT	14-49	5×10^9	32	84	0%
Marmoset							
CC04 (f)	0.35/6	CT	14-49	5×10^9	2.7	86	98.3%
CC05 (m)	0.35/7	CT	14-49	5×10^9	3.1	83	99.6%
CC02 (m)	0.33/3	CT	14-49	5×10^9	2.8	77	0%
EW03 (f)	0.35/5	EDC	14-49	5×10^9	100	84	100%
EW09 (m)	0.37/5	EDC	17-24	2×10^9	100	84	100%
